# Radical hysterectomy for FIGO stage I–IIB adenocarcinoma of the uterine cervix

**DOI:** 10.1038/sj.bjc.6605048

**Published:** 2009-04-28

**Authors:** T Kasamatsu, T Onda, M Sawada, T Kato, S Ikeda, Y Sasajima, H Tsuda

**Affiliations:** 1 Division of Gynecology, National Cancer Center Hospital, Tokyo, Japan; 2 Division of Diagnostic Pathology, National Cancer Center Hospital, Tokyo, Japan

**Keywords:** radical hysterectomy, adenocarcinoma, uterine cervix, FIGO stage I–IIB

## Abstract

A retrospective analysis was carried out to identify risk factors for survival and relapse in patients with FIGO stage I–IIB cervical adenocarcinoma (AC), who underwent radical hysterectomy, and to compare outcome and spread pattern with those of squamous cell carcinoma (SCC). One hundred and twenty-three FIGO stage I–IIB patients with AC and 455 patients with SCC, who all underwent primary radical hysterectomy, were reviewed. Among the patients with AC, Cox model identified tumour size (95% CI: 1.35–30.71) and node metastasis (95% CI: 5.09–53.44) as independent prognostic factors for survival, and infiltration to vagina (95% CI: 1.15–5.76) and node metastasis (95% CI: 6.39–58.87) as independent prognostic factors for relapse. No significant difference was found in survival or relapse between the AC and SCC groups, after adjusting for other clinicopathological characteristics using Cox model. No significant difference was found in the positive rates of lymph nodes or location of initial failure sites between the two groups, but ovarian metastatic rate was significantly higher in patients with pathologic stage IIB AC (*P*=0.02). Positive node is a common independent prognostic factor for survival and relapse of patients with AC. FIGO stage I–IIB patients with AC or SCC, who underwent radical hysterectomy, have similar prognosis and spread pattern, but different ovarian metastasis rates.

At present, standard treatment options for patients with invasive carcinoma of the uterine cervix are as follows: radical hysterectomy followed by adjuvant radiotherapy or primary radiotherapy with concurrent cisplatin-containing chemotherapy, for the patients with the International Federation of Gynecology and Obstetrics (FIGO) stage IB–IIA disease, with equivalent results; and primary radiotherapy with concurrent chemotherapy for the patients with FIGO stage IIB–IVA disease. These therapeutic strategies have been widely accepted. On the other hand, approximately 85% of the patients with carcinoma of the uterine cervix have squamous lesions, and most of the remaining 10% have adenocarcinoma (AC) lesions (Benedet *et al*, 2003). To our knowledge, no practice guideline has referred to the treatment option based on the difference of histological types between AC and squamous cell carcinoma (SCC) It is not clear whether these histological types influence outcome or spread pattern, and there is still controversy, as conflicting results have appeared in the literature because of potential limitations of small cohorts of patients with AC. The question whether standard treatment for patients with SCC is also suitable for patients with AC remains unanswered. Additionally, over the last decade, the proportion of AC relative to SCC has doubled, and the rate of AC per population at risk has also increased (Smith *et al*, 2000). To establish a framework for designing therapeutic strategies, the present retrospective study was undertaken firstly to clarify the clinicopathological features of the surgically treated patients with common type of AC and to identify prognostic factors. Secondly, comparisons of outcomes and spread pattern were made between patients with AC and SCC. Our study will support the design of therapeutic strategies, including surgery, radiotherapy, and chemotherapy, that will be more suitable for different disease types.

## Patients and methods

### Patients

We reviewed the medical records and pathological materials that had been obtained from 1189 patients with the FIGO stage IB–IVA invasive carcinoma of the uterine cervix, who were treated at the Gynecology Division of the National Cancer Center Hospital, Tokyo, Japan, between 1984 and 2003. This study included patients who met the following criteria: the patients had (a) common histological subtypes of endometrioid AC and endocervical type AC or (b) SCC; the patients had FIGO stage I–IIB disease; and the patients underwent primary surgery consisting of radical hysterectomy with pelvic lymphadenectomy. Patients who received preoperative chemotherapy or radiotherapy were excluded. Patients with uncommon histological subtypes of AC (adenoma malignum, villoglandular, intestinal type, clear cell, serous, or mesonephric AC), and those who had other epithelial carcinoma (adenosquamous, glassy cell, adenoid cystic, adenoid basal, small cell, or undifferentiated carcinoma) were also excluded. Patients with SCC were included in this study for critical comparison of spread pattern, recurrence, and survival.

All of the patients were staged according to the FIGO staging system. Postoperative pathological classification was carried out according to the International Union Against Cancer (UICC) TNM classification of malignant tumours. Histological typing was evaluated according to the criteria of the World Health Organization International Histological Classification of Tumours.

### Treatment

The radicality of hysterectomy in this study corresponded to classes III and IV of the Piver–Rutledge classification (Piver *et al*, 1974). In patients with pelvic lymph node metastasis (pT1bN1, pT2aN1, or pT2bN1) or parametrial involvement (pT2b) proven by pathological examination following surgery, adjuvant postoperative irradiation to the whole pelvis was administered. A daily dose of 2 Gy, five fractions a week, was given using a linear accelerator. The total dose for the whole pelvis was 50 Gy with an opposed anterior and posterior field, or a four-field anterior–posterior and lateral technique. All slides of resected specimens were examined by three to four pathologists independently, and a consensus diagnosis was reached. The categories of lymph–vascular space invasion were defined as follows: after the examination of all slides of tumour tissues, they were categorised as none (no foci of lymph–vascular space invasion), few (1–2 foci), several (3–5 foci), or many (more than 5 foci of lymph–vascular space invasion).

Following the primary treatment, asymptomatic patients underwent pelvic examination, Pap smear, chest radiograph, ultrasonography, and determination of serial tumour markers (SCC, CEA) every 4–6 months. Symptomatic patients underwent the appropriate examination where indicated using computed tomography (CT) and magnetic resonance imaging (MRI).

### Statistical methods

Survival and relapse-free survival (RFS) curves were obtained by the Kaplan–Meier method and the survival curves were compared by non-parametric survival analysis (log-rank test). A *P*-value of <0.05 was considered to indicate statistical significance. Variables that showed a significant association with survival were included in multivariate analysis based on the Cox proportional–hazard model with a stepwise method (forward selection). A *P*-value of <0.05 was adopted as inclusion criteria, and a *P*-value of >0.10 was adopted as exclusion criteria for the forward selection. For categorical variables, Fisher's exact test, or *χ*^2^-test was used. Patients who died of other causes were included as deaths in the survival analysis. Follow-up continued through to December 2007. All statistical analyses were carried out with the statistical software package SPSS for Windows (version 11.0J; SPSS Inc., Chicago, IL, USA).

## Results

### Patient characteristics

In all, 123 patients with cervical AC and 455 patients with cervical SCC met the study criteria. The characteristics of the patients are summarised in Table 1. Median age of the patients with AC was 48 years (range: 29–71) and median age of the patients with SCC was 47 years (range: 22–73). Following surgery, 226 patients received adjuvant therapy. Majority of the patients received standard adjuvant therapy, which was irradiation to the whole pelvis, but five patients with AC who refused radiotherapy received chemotherapy. Among the seven patients with positive surgical margin, five patients received radiotherapy to control microscopic residual tumour in the vaginal stump, and the remaining two patients refused postoperative treatment.

All 578 patients were followed for 1–288 months, including until death, and the median follow-up period was 93 months. The details with regard to recurrent sites are not available for one patient, who died of the disease at another hospital.

### Survival

The overall survival and the RFS of the patients with AC were assessed by log-rank test in two clinical and nine pathological subgroups (Table 2). In these, multivariate analysis testing for differences in survival among statistically significant subgroups of FIGO stage, tumour size, depth in cervical wall, number of positive nodes, degree of lymph–vascular invasion, pathological parametrial involvement, infiltration to vagina, ovarian metastases, and histological grade was carried out. The Cox model with a forward stepwise method identified that tumour size (over 40 mm) and number of positive nodes were independent prognostic factors for overall survival. Similarly, the RFS was also assessed in the subgroups according to age in addition to the same parameters as overall survival. The Cox model showed that infiltration to vagina and number of positive nodes were independent prognostic factors for relapse (Table 3). Among the patients who recurred, one patient with pT1b-2N1 disease survived after recurrence.

Comparison of survival rate was made between the patients with AC and those with SCC according to UICC pathological stage. Among the patients with AC, the cumulative 5-year survival rates of the patients with pathological stage pT1b, pT2a, and pT2b diseases were 89, 92, and 38%, respectively. Among the patients with SCC, the 5-year survival rates of the patients with pT1b, pT2a, and pT2b diseases were 89, 89, and 62%, respectively. Univariate analysis revealed no significant difference in survival between patients with AC and SCC (log-rank, *P*=0.640 in the patients with pT1b disease, *P*=0.317 in pT2a disease, and *P*=0.074 in pT2b disease). Similarly, among the patients with AC, the RFS rates at 36 months of the patients with pT1b, pT2a, and pT2b diseases were 91, 100, and 38%, respectively, compared with 91, 91, and 61% of the patients with SCC, respectively. There were no significant differences in relapse between patients with AC and SCC (log-rank, *P*=0.860 in the patients with pT1b disease, *P*=0.227 in pT2a, and *P*=0.137 in pT2b). To adjust for other clinicopathological characteristics, the Cox model was used for survival and RFS analyses among the subgroups according to age, postoperative therapy, tumour size, depth in cervical wall, lymph node status, LVS invasion, ovarian metastasis, and histological types (AC or SCC). The Cox model-adjusted clinicopathological characteristics showed no significant difference in survival or relapse between the AC and SCC groups (Tables 4 and 5). Histological type was not shown to be an independent factor of survival or relapse at any pathological stage.

### Spread pattern and failure sites

Among the 123 patients with AC, the positive rate of pelvic lymph node metastasis at the initial surgery was 16% (14 of 88) of the patients with pT1b disease, 14% (2 of 14) of the patients with pT2a disease, and 76% (16 of 21) of the patients with pT2b disease, compared with 17% (38 of 229), 21% (23 of 112), and 75% (85 of 114), respectively, of the patients with SCC. There were no significant differences in the positive node rates between AC and SCC groups (Fisher's exact test, *P*=1.000 in the patients with pT1b disease, *P*=0.735 in the patients with pT2a disease, and *P*=1.000 in the patients with pT2b disease). With regard to the paraaortic lymph node status, in the AC group, no enlarged paraaortic nodes were found during the operation. Further, common iliac node metastasis was proven in 10 (31.3%, 10 of 32) patients with positive pelvic lymph nodes. In the SCC group, 41 (28.1%, 41/146) patients with positive pelvic lymph nodes had common iliac node metastasis. Of these, eight patients had paraaortic lymph node metastasis, which was proven histopathologically, and enlarged paraaortic lymph nodes were not found during the operation in the remaining patients.

Among the patients with AC, ovarian metastasis was found in one patient (1.1%, 1 of 87) with pT1b disease, no patient with pT2a disease, and five patients (23.8%, 5 of 21) with pT2b disease. Among the patients with SCC, ovarian metastasis was found in one patient (0.4%, 1 of 228) with pT1b disease, two patients (1.7%, 2 of 112) with pT2a disease, and three patients (2.6%, 3 of 114) with pT2b disease. Significant difference was found in ovarian metastatic rate between AC and SCC groups with pT2b disease (Fisher's exact test, *P*=0.477 in the patients with pT1b disease, *P*=1.000 in the patients with pT2a disease, and *P*=0.002 in the patients with pT2b disease). From the viewpoint of clinical FIGO stage, the ovarian metastatic rates in the AC group were 3.2% (3 of 95) of patients with FIGO stage IB, 0% (0 of 5) with FIGO stage IIA, and 13.6% (3 of 22) with FIGO stage IIB. Similarly, in the SCC group, the ovarian metastatic rates were 0.4% (1 of 274) of patients with FIGO stage IB, 1.9% (1 of 51) with FIGO stage IIA, and 3.1% (4 of 129) with FIGO stage IIB.

Among the 123 patients with AC, 27 patients (22%) suffered tumour recurrence. Of these, the initial failure sites were located inside the pelvis in 10 (38%) patients, outside the pelvis in 15 (58%) patients, and both inside and outside the pelvis in 1 (4%) patient. Unknown site was in one patient. Among the 455 patients with SCC, 89 (20%) patients suffered recurrence. The initial failure sites were located inside the pelvis in 28 (32%) patients, outside the pelvis in 57 (64%) patients, and both inside and outside the pelvis in 4 (4%) patients. No significant difference was found in location of initial failure sites between AC and SCC groups (*χ*^2^-test, *P*=0.288).

Of all initial failure sites located outside the pelvis in the 15 patients with AC who recurred, the most frequent sites were distant nodes (48%) and peritoneal spread (48%), followed by the lung (8%) and bone (8%). In the 57 patients with SCC, the most frequent sites located outside the pelvis were distant nodes (48%), followed by the lung (25%), bone (16%), the liver (9%), and peritoneal spread (2%).

## Discussion

Since 1980, three studies using multivariate analysis have reported prognostic factors in patients with cervical AC who underwent radical hysterectomy followed by adjuvant radiotherapy. Matthews *et al* (1993) showed that only nodal positivity was the major prognostic factor for survival in 79 patients with clinical stage I disease. Irie *et al* (2000) also reported the same result in 57 patients with FIGO stage I–IIB disease (Irie *et al*, 2000). Ishikawa *et al* (1999) reported that the clinical stage, the presence of nodal metastasis, lymph–vascular space invasion, and tumour size were independent risk factors in 193 patients with FIGO stage I–IV disease, and number of positive nodes and tumour size were independent risk factors in survival and relapse among patients with FIGO stage I disease. These results were dependent on characteristics of the enrolled patients because of their small cohorts. These three reports included several pathological subtypes of adenosquamous cell carcinoma, clear cell carcinoma, or subtypes of which details were not mentioned. As for the prognostic significance of pathological subtype, conflicting results have appeared in the literature (Korhonen, 1984; Alfsen *et al*, 2001; Lea *et al*, 2003). In this study, we employed only ordinary subtypes of AC of the uterine cervix to simplify the analysis. Nonetheless, based on the literature and our data, it is reasonably certain that nodal positivity is recognised as a common independent adverse prognostic factor for survival and relapse of the patient with FIGO stage I–IIB disease who underwent radical hysterectomy. Tumour size was an independent adverse factor only for survival, and vaginal infiltration was an independent risk factor only for relapse. The following is our explanation regarding these issues. In contrast to SCC, cervical AC arises from the endocervix. The lesion expands into the endocervix and characteristically creates a bulky tumour in the cervical canal, after which it invades the vagina directly. In this study, 66% (19 of 29) of the patients in the AC group with vaginal infiltration had large-sized tumours (>40 mm), compared with 42% (74 of 175) in the SCC group. There may be multicollinearity between tumour size and vaginal invasion as assessed by the Cox model.

In this study, there was no significant difference in survival or relapse, after adjusting for other clinicopathological characteristics, between the AC and SCC groups at any pathological stage. One limitation of this study was that it was a retrospective study with a limited number of statistical events, thus, it was difficult to evaluate power calculation statistically. Grisaru *et al* (2001) reported that 799 surgically treated patients with FIGO stage IA–IB disease of AC or SCC had a similar prognosis. Look *et al* (1996) and Lee *et al* (2006) reported same results in 749 and 60 patients with FIGO stage IB disease, respectively. A similar finding was noted in a study from Fregnani *et al* (2008), in which 238 FIGO stage IB–IIA patients with AC or SCC were assessed. In these reports, radical hysterectomy followed by adjuvant radiotherapy was employed as a treatment modality. Nakanishi *et al* (2000) showed that histology of AC was an independent significant risk factor for survival and relapse in pathologic stage IB (pT1b) patients who underwent radical hysterectomy. They also reported that the prognosis of patients with AC was poorer than that of patients with SCC in the presence of lymph node metastasis, whereas the prognosis was equivalent when there was no metastasis. Although further investigation is still needed, considering our data and the present literature, radical hysterectomy followed by adjuvant radiotherapy is still considered the standard treatment option for early stage cervical AC with equivalent results for cervical SCC. However, there is still controversy regarding the advanced stage disease, that is, FIGO stage IIB. Radiotherapy has been employed as the standard treatment option for FIGO stage IIB disease in many countries, and radical hysterectomy has been adopted for stage IB–IIA disease (Suprasert *et al*, 2005). The 25th FIGO annual report from 93 centres throughout the world reported that 72% (2320 of 3233) of the patients with stage IIB disease were treated with radiotherapy (Benedet *et al*, 2003). Accordingly, few reports discuss the radical hysterectomy for patients with FIGO stage IIB cervical AC. On the other hand, it was noted that although primary radiotherapy is effective for patients with small volume stage IB AC lesions with equivalent results for SCC lesions, it does not appear to be sufficient for patients with advanced stage II or large tumour size AC lesions (Eifel *et al*, 1990, 1995; Berek, 1995). This study included the patients with stage IIB disease who also underwent radical hysterectomy. In our institute, 88% of the patients with stage IIB AC underwent radical hysterectomy of classes III and IV, and the remaining 12% were treated by primary radiotherapy during the study period. Consequently, no significant difference was found in survival or relapse between the surgically treated patients with pT2b AC and SCC lesions in this study. Considering the above facts, radical hysterectomy may be a treatment of choice for stage IIB disease in cases of AC in contrast to SCC.

The incidence of ovarian metastasis of AC lesion was significantly higher than that of SCC of pT2b lesion in this study. Similarly, Nakanishi *et al* (2001) reported that the incidences of metastasis of FIGO stages IB and IIB AC lesions were 4.0 (7 of 178) and 14.8% (4 of 27), respectively, whereas the incidences of FIGO stages IB and IIB SCC lesions were 0.5 (3 of 614) and 4.0% (7 of 175), respectively. When pathological parametrial invasion was present, the incidence increased from 3 to 25.6%. In their report, using a logistic analysis with clinicopathological variables revealed that the presence of pathological endometrial invasion, lymph node metastasis, and pathological parametrial invasion were the significant variables associated with ovarian metastasis of AC lesion. Ovarian preservation should not be recommended in cases of AC except for very early lesions.

In this study, there were no significant differences in positive rates of pelvic lymph node between AC and SCC groups at any pathological stage. In the reported literature, no differences were found in positive lymph node rates of FIGO stage IB disease, and positive rates were approximately 5–15% (Look *et al*, 1996; Irie *et al*, 2000; Lee *et al*, 2006). Few reported literature have discussed lymph node status of advanced stage, that is, FIGO stage IIB. Irie *et al* (2000) reported a higher positive rate of lymph node in patients with AC than in those with SCC of FIGO stage II disease (57.1 *vs* 26.9%). This discrepancy is probably because of the number of patients, the difference in histological subtypes of AC, and/or the difference between clinical and pathological stages.

No differences were noted in initial failure sites among the patients in the two histological groups, which were classified into inside and outside the pelvis in this study. A similar finding was reported in a study by Grisaru *et al* (2001), in which 100 patients with FIGO stage IA–IB disease were treated in the same manner. Of the distribution of the distant metastatic sites, peritoneal spread was more frequent in AC (48 *vs* 2%) in our study. Drescher *et al* (1989) reported that disseminated peritoneal involvement was twice as frequent in patients with AC from 21 autopsy findings. Although the data are not yet sufficient, disseminated peritoneal spread might be a characteristic of AC of the uterine cervix.

In conclusion, number of positive nodes is a common independent prognostic factor for survival and relapse among the FIGO stage I–IIB patients with ordinary types of AC who underwent radical hysterectomy followed by adjuvant radiotherapy. Surgically treated patients with AC or SCC have a similar prognosis and spread pattern, but not the ovarian metastasis rate.

## Figures and Tables

**Table 1:** Patient characteristics

	**Adenocarcinoma**	**Squamous cell carcinoma**	
	**No. (%)**	**No. (%)**	***P*-value**
*FIGO stage*			
IB	96 (78)	275 (60)	0.021
IIA	5 (4)	51 (11)	
IIB	22 (18)	129 (29)	
			
*Pathological stage*			
pT1b	88 (72)	229 (50)	<0.001
pT2a	14 (11)	112 (25)	
pT2b	21 (17)	114 (25)	
			
*Tumour size (mm)*			
≦20	37 (30)	92 (20)	0.024
21–40	46 (37)	222 (49)	
>40	40 (33)	141 (31)	
			
*Depth in cervical wall*			
<1/3	34 (28)	68 (15)	0.002
1/3–2/3	31 (25)	107 (23)	
>2/3	58 (47)	280 (62)	
			
*Number of positive pelvic lymph nodes*			
None	91 (74)	309 (68)	0.329
1–4	25 (20)	104 (23)	
≧5	7 (6)	42 (9)	
			
*Lymph–vascular space invasion*			
None	53 (43)	102 (22)	<0.001
Few	32 (26)	149 (33)	
Several	27 (22)	110 (24)	
Many	11 (9)	94 (21)	
			
*Infiltration to vagina*			
No	94 (76)	280 (62)	0.003
Yes	29 (24)	175 (38)	
			
*Ovarian metastases*			
Negative	116 (94)	448 (98.5)	0.024
Positive	6 (5)	6 (1.3)	
Not resected	1 (1)	1(0.2)	
			
*Surgical margin*			
Free	121 (98)	450 (99)	0.644
Close or involved	2 (2)	5 (1)	
			
*Postoperative therapy*			
Not carried out	94 (76)	253 (56)	<0.001
Radiotherapy	24 (20)	202 (44)	
Chemotherapy	5 (4)	0	

FIGO=the International Federation of Gynecology and Obstetrics.

**Table 2:** Overall survival and disease-free survival in 123 patients with adenocarcinoma

	**No.**	**5-year survival**	**Log-rank**	**RFS at 36 (months)^a^**	**Log-rank**
	**(%)**	**(%)**	***P*-value**	**(%)**	***P*-value**
*Age*					
≦48	67 (54)	84	0.091	85	0.017
>48	56 (46)	77		80	
					
*FIGO stage*					
IB	96 (78)	86	0.004	89	0.001
IIA	5 (4)	53		60	
IIB	22 (18)	59		59	
					
*Tumour size (mm)*					
≦20	37 (30)	95	<0.001	97	<0.001
21–40	46 (37)	91		91	
>40	40 (33)	55		59	
					
*Depth in cervical wall*					
<1/3	34 (28)	97	0.004	100	0.001
1/3–2/3	31 (25)	87		87	
>2/3	58 (47)	67		87	
					
*Number of positive nodes*					
None	91 (74)	91	<0.001	93	<0.001
1–4	25 (20)	60		63	
≧5	7 (6)	14		14	
					
*LVS invasion* b					
None	53 (43)	98	<0.001	98	<0.001
Few	32 (26)	81		84	
Several	27 (22)	56		61	
Many	11 (9)	55		55	
					
*Parametrial invasion*					
Negative	102 (83)	89	<0.001	92	<0.001
Positive	21 (17)	38		43	
					
*Infiltration to vagina*					
No	94 (76)	87	<0.001	89	<0.001
Yes	29 (24)	61		62	
					
*Ovarian metastases*					
Negative	94 (95)	84	<0.001	85	<0.001
Positive	6 (5)	17		20	
					
*Histological subtype*					
Mucinous	73 (59)	75	0.543	78	0.180
Endometrioid	50 (41)	88		90	
					
*Histological grade*					
Grade 1	86 (70)	85	<0.001	86	<0.001
Grade 2	23 (19)	87		86	
Grade 3	14 (11)	47		56	

FIGO=the International Federation of Gynecology and Obstetrics; LVS=lymph–vascular space; RFS=relapse-free survival.

a RFS at 36 months.

b LVS invasion.

**Table 3:** Multivariate analysis of prognostic factors for OS and RFS in 123 patients with AC (with a stepwise method, forward selection)^a^

	**OS**			**RFS**		
	**HR**	**95% CI** b	***P*-value**	**HR**	**95% CI**	***P*-value**
*Infiltration to vagina*						
No	—	—	—	1.00		
Yes	—	—	—	2.58	1.15–5.76	0.020
						
*Tumour size (mm)*						
≦20	1.00					
21–40	2.25	0.45–11.29	0.323	—	—	—
>40	6.44	1.35–30.71	0.019	—	—	—
						
*Number of positive nodes*						
None	1.00			1.00		
1–4	2.63	1.13–6.10	0.024	3.86	1.58–9.41	0.003
>5	16.50	5.09–53.44	<0.001	19.39	6.39–58.87	<0.001

AC=adenocarcinoma; CI=confidence interval; FIGO=the International Federation of Gynecology and Obstetrics; HR=hazard ratio; LVS=lymph–vascular space; OS=overall survival; RFS=relapse-free survival.

a The analysis was adjusted for FIGO stage, tumour size, depth in cervical wall, number of positive node, degree of LVS invasion, pathological parametrial involvement, infiltration to vagina, ovarian metastases, and histological grade.

b 95% confidence interval.

**Table 4:** Multivariate analysis of prognostic factors for OS in 123 patients with AC and 455 patients with SCC (with a stepwise method, forward selection)

	**PT1b**		**pT2a**		**pT2b**	
	**HR**	**95% CI**	**HR**	**95% CI**	**HR**	**95% CI**
*Age*						
≦48	1.00		*P*>0.10^a^		*P*>0.10	
>48	2.15	1.61–3.98				
						
*Postoperative therapy*						
Carried out	*P*>0.10		*P*>0.10		1.00	
Not carried out					2.44	1.38–4.32
						
*Tumour size (mm)*						
≦20	1.00		*P*>0.10		*P*>0.10	
21–40	2.37	0.95–5.90				
>40	4.00	1.48–10.75				
						
*Number of positive nodes*						
None	1.00		1.00		1.00	
1–4	1.71	0.80–3.68	1.99	0.54–7.29	1.66	0.88–3.16
>5	7.76	2.70–22.27	46.38	3.26–658.60	5.25	2.65–10.39
						
*Ovarian metastases*						
Negative	*P*>0.10		*P*>0.10		1.00	
Positive					2.66	1.00–7.04
						
*Depth in cervical wall*						
<1/3	*P*>0.10		*P*>0.10		*P*>0.10	
1/3–2/3						
>2/3						
						
*LVS invasion*						
None	*P*>0.10		*P*>0.10		*P*>0.10	
Few						
Several						
Many						
						
*Histological type*						
SCC	*P*>0.10		*P*>0.10		*P*>0.10	
AC						

AC=adenocarcinoma; CI=confidence interval; FIGO=the International Federation of Gynecology and Obstetrics; HR=hazard ratio; LVS=lymph–vascular space; OS=overall survival; SCC=squamous cell carcinoma.

a *P*>0.10 was exclusion criteria for the stepwise, forward selection.

**Table 5:** Multivariate analysis of prognostic factors for RFS in 123 patients with AC and 455 patients with SCC (with a stepwise method, forward selection)

	**pT1b**		**pT2a**		**pT2b**	
	**HR**	**95% CI**	**HR**	**95% CI**	**HR**	**95% CI**
*Age*						
≦48	*P*>0.10^a^		*P*>0.10		*P*>0.10	
>48						
						
*Postoperative therapy*						
Done	*P*>0.10		*P*>0.10		1.00	
Not done					3.14	1.75–5.64
						
*Tumour size (mm)*						
≦20	1.00		*P*>0.10		*P*>0.10	
21–40	2.63	0.87–7.94				
>40	7.25	2.34–22.48				
						
*Number of positive nodes*						
None	1.00		1.00		1.00	
1–4	1.75	0.79–3.86	3.63	1.15–11.47	2.04	0.93–4.93
>5	8.30	2.83–24.33	13.85	2.83–67.68	6.62	3.00–14.64
						
*Ovarian metastases*						
Negative	*P*>0.10		*P*>0.10		*P*>0.10	
Positive						
						
*Depth in cervical wall*						
<1/3	*P*>0.10		*P*>0.10		*P*>0.10	
1/3–2/3						
>2/3						
						
*LVS invasion*						
None	*P*>0.10		*P*>0.10		*P*>0.10	
Few						
Several						
Many						
						
*Histological type*						
SCC	*P*>0.10		*P*>0.10		*P*>0.10	
AC						

AC=adenocarcinoma; CI=confidence interval; FIGO=the International Federation of Gynecology and Obstetrics; HR=hazard ratio; LVS=lymph–vascular space; OS=overall survival; RFS=relapse-free survival; SCC=squamous cell carcinoma.

a *P*>0.10 was exclusion criteria for the stepwise, forward selection.
